# First detection of highly pathogenic avian influenza virus in Norway

**DOI:** 10.1186/s12917-021-02928-4

**Published:** 2021-06-12

**Authors:** Knut Madslien, Torfinn Moldal, Britt Gjerset, Sveinn Gudmundsson, Arne Follestad, Elliot Whittard, Ole-Herman Tronerud, Katharine Rose Dean, Johan Åkerstedt, Hannah J. Jørgensen, Carlos G. das Neves, Grim Rømo

**Affiliations:** 1 Norwegian Veterinary Institute, Oslo, Norway; 2 Norwegian Institute of Nature Research, Trondheim, Norway; 3 Animal & Plant Health Agency, Surrey, UK; 4 Norwegian Food Safety Authority, Oslo, Norway

**Keywords:** *Anseriformes*, *Charadriiformes*, HPAI, H5N8, Surveillance, Measures

## Abstract

**Background:**

Several outbreaks of highly pathogenic avian influenza (HPAI) caused by influenza A virus of subtype H5N8 have been reported in wild birds and poultry in Europe during autumn 2020. Norway is one of the few countries in Europe that had not previously detected HPAI virus, despite widespread active monitoring of both domestic and wild birds since 2005.

**Results:**

We report detection of HPAI virus subtype H5N8 in a wild pink-footed goose (*Anser brachyrhynchus*), and several other geese, ducks and a gull, from south-western Norway in November and December 2020. Despite previous reports of low pathogenic avian influenza (LPAI), this constitutes the first detections of HPAI in Norway.

**Conclusions:**

The mode of introduction is unclear, but a northward migration of infected geese or gulls from Denmark or the Netherlands during the autumn of 2020 is currently our main hypothesis for the introduction of HPAI to Norway. The presence of HPAI in wild birds constitutes a new, and ongoing, threat to the Norwegian poultry industry, and compliance with the improved biosecurity measures on poultry farms should therefore be ensured. [MK1]Finally, although HPAI of subtype H5N8 has been reported to have very low zoonotic potential, this is a reminder that HPAI with greater zoonotic potential in wild birds may pose a threat in the future. [MK1]Updated with a sentence emphasizing the risk HPAI pose to poultry farms, both in the Abstract and in the Conclusion-section in main text, as suggested by Reviewer 1 (#7).

## Background

Influenza A viruses are common in wild birds worldwide, especially in species within the orders of *Anseriformes* (ducks, geese, and swans) and *Charadriiformes* (gulls, terns, and shorebirds) [1].

Several outbreaks of highly pathogenic avian influenza (HPAI) have affected domestic poultry and wild birds in Europe in recent years, and Influenza A virus of subtypes H5N1 and H5N8 have predominated [2]. Viruses from the H5N8 Gs/GD clade 2.3.4.4 Group B, originally from Asia, were found to be highly pathogenic in geese in 2016/2017 [3]. Between August 15th and December 7th 2020, 561 detections of HPAI virus have been reported in 15 EU/EEA countries and the UK, with subtype H5N8 (*n* = 518) as the most reported subtype, followed by A(H5N5) (*n* = 17) and A(H5N1) (*n* = 6) [4]. Mainly wild birds (*n* = 510), such as barnacle goose (*Branta leucopsis*), greylag goose (*Anser anser*), Eurasian wigeon (*Mareca penelope*) and mallard (*Anas platyrhynchos*) were affected, but there have also been a few outbreaks in poultry (*n* = 43) [4].

Norway is one of the few countries in Europe that had not previously detected HPAI virus, despite widespread active monitoring of both domestic and wild birds since 2005 [2, 5]. Prevalence of influenza A virus in ducks and gulls varied between 5.7 and 18.4 % in the period 2005–2019 in Norway, all being low pathogenic avian influenza (LPAI) virus [5]. All influenza A positive samples from the monitoring program are tested for the presence of subtype H5 and H7. Out of the 34 (6.7 %) influenza A positive samples in 2019, one sample was H5 positive, whereas no samples were H7 positive. The H5 positive sample was collected from a mallard, and was negative for neuraminidase subtype N1 [5]. The sample was not tested for other neuraminidase subtypes, as this is not specified in the mandate of the active monitoring program. However, sequencing of the HA genes identified the virus as LPAI, which precludes the possible presence of HPAI H5N8 as early as 2019.

## Results

**Fig. 1:**
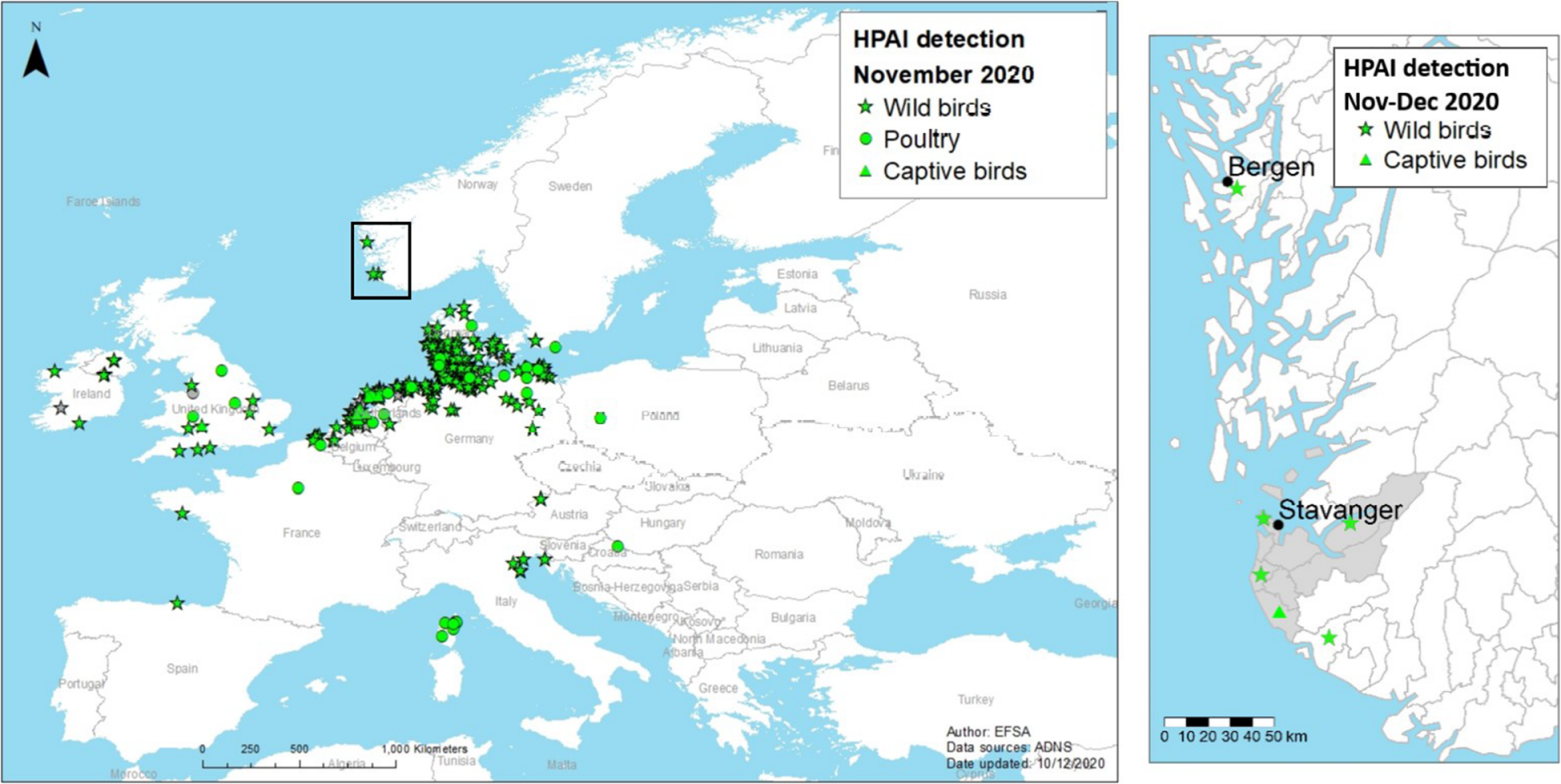
**Left panel**. Detections of Influenza A(H5N8) virus reported in wild birds (stars), poultry (circles), and captive birds (triangles), by EU/EEA countries in November 2020 (*n* = 520). The black square shows the region of south Norway. **Left panel** reprinted with permission from EFSA (original in 10.2903/j.efsa.2020.6379). **Right panel**. HPAI detections in wild and captive birds in Southern Norway for November and December 2020. Black dots show the cities of Bergen and Sandnes. Grey shading highlights the municipalities that are in the traditional district of Jæren in Rogaland county

On November 27th 2020, the Norwegian Veterinary Institute (NVI) notified the Norwegian Food Safety Authority (NFSA) of a confirmed case of HPAI virus subtype H5N8 in a wild pink-footed goose (*Anser brachyrhynchus*) from Sandnes municipality, in Rogaland County (Fig. 1). The goose was found in a diseased state and died on the 22nd of November 2020. Already on November 19th 2020, prior to the first detection of HPAI, a request for increased vigilance to report sick or dead wild birds had been communicated to the public and bird watchers, through the national media and on the websites of the NFSA and NVI. This increased awareness was crucial for the notification of the first HPAI-positive, pink-footed goose to the NFSA, since HPAI could not be ruled out by the person who found the diseased bird. Despite previous reports of LPAI, this constitutes the first detection of HPAI in Norway.

As an immediate response to the detection of HPAI in Norway, the NFSA introduced a high-risk area in line with Commission Implementing Decision (EU) 2018/1136 of August 10th 2018 on risk mitigation and reinforced biosecurity measures and early detection systems in relation to the risks posed by wild birds for the transmission of highly pathogenic avian influenza viruses to poultry. This included a housing order for poultry and birds kept in captivity in the coastal municipalities from Rogaland county in the southwest to the Swedish border in the southeast. As a result of multiple detections of HPAI in two counties in the two weeks following the initial detection (Table 1), the high-risk area was extended to Southern Norway south of Nordland county on December 7th 2020 [6]. Poultry farmers were also informed of the risk from wild birds and encouraged to strengthen their biosecurity measures, and to immediately submit birds for AI analysis in the event of increased mortality, decrease in egg-production or decrease in intake of water and feed in their flocks.

**Table 1:** All birds positive for HPAI virus of subtype H5N8 in Norway by 22^th^ December 2020

Sampling	Municipality	County	Species	Active/passive surveillance
14/11/2020	Klepp	Rogaland	Eurasian wigeon (*Mareca penelope*)	Active
14/11/2020	Klepp	Rogaland	Eurasian wigeon (*Mareca penelope*)	Active
14/11/2020	Klepp	Rogaland	Eurasian wigeon (*Mareca penelope*)	Active
15/11/2020	Klepp	Rogaland	Eurasian wigeon (*Mareca penelope*)	Active
24/11/2020	Sandnes	Rogaland	Pink-footed goose (*Anser brachyrhynchus*)^**a**^	Passive
28/11/2020	Klepp	Rogaland	Pink-footed goose (*Anser brachyrhynchus*)	Active
30/11/2020	Bergen	Vestland	Great black-backed gull (*Larus marinus*)	Passive
30/11/2020	Hå	Rogaland	Domestic turkey (*Meleagris gallopavo f. domestica*)	Passive
02/12/2020	Hå	Rogaland	Domestic fowl (*Gallus gallus domesticus*)	Passive
02/12/2020	Hå	Rogaland	Domestic fowl (*Gallus gallus domesticus*)	Passive
02/12/2020	Hå	Rogaland	Domestic fowl (*Gallus gallus domesticus*)	Passive
03/12/2020	Randaberg	Rogaland	Eurasian wigeon (*Mareca penelope*)	Passive
10/12/2020	Eigersund	Rogaland	European herring gull (*Larus argentatus*)	Passive
17/12/2020	Evje og Hornes	Agder	Barnacle goose (*Branta leucopsis*)	Passive

^a^Index case

On December 14th 2020, the NFSA issued regulation for an immediate ban of waterfowl hunting in Southern Norway until further notice [7]. The main reason for introducing this ban was to avoid unnecessary disturbance to the birds and to prevent potentially infected waterfowl from moving out of infected areas. Hunters generally shoot healthy birds, but occasionally they may shoot sub-clinically infected birds or those early in the incubation period. Handling and processing these infected birds may pose a potential risk to poultry in areas with a high density of farms (Fig. 1).

Sequencing of the cleavage site only, performed by NVI, showed that all HPAI virus detected in Norway belongs to the clade 2.3.4. Genetic characterization of the complete genome of one H5N8 virus identified in Norway in January 2021 was performed by EU/OIE/National Reference Laboratory for Avian Influenza and Newcastle Disease. Phylogenetic analysis of the HA gene revealed that the virus belongs to the clade 2.3.4.4B and are closely related to other H5N8 HPAI viruses detected in wild birds, poultry and turkey across Europe and central Asia during winter of 2020–2021. Sequence comparisons demonstrated high identity for all gene segments (99.4–99.8 %) with wild bird subtypes H5N8 and H5N5 sequences from neighbouring countries including the United Kingdom, the Netherlands, Germany, Denmark, Belgium and Russia with the most recent common ancestor being A/chicken/Iraq/1/2020 (H5N8).

As of the 22nd of December 2020, HPAI virus of subtype H5N8 has been detected in ten wild and four captive birds, all in Southern Norway (Table 1).

A great black-backed gull (*Larus marinus*) was observed sick for a couple of days in Bergen municipality, Vestland county, and died on the 29th of November 2020. Bergen is around 170 km north of Sandnes (Fig. 1; Table 1). Additionally, another A(H5N8) positive pink-footed goose was discovered in Klepp municipality, Rogaland, about 15 km from the index case; the goose displayed no clinical signs and was shot during regular hunting.

A captive turkey (*Meleagris gallopavo f. domestica*) and three roosters (*Gallus gallus domesticus*) were found dead in late November and early December, respectively, in a publicly operated bird park in Hå municipality, about 15 km south of Sandnes (Fig. 1; Table 1).

Four Eurasian wigeons shot during the regular hunting season in mid-November in Klepp municipality were positive for HPAI of subtype H5N8 (Table 1). Samples from these birds were submitted to NVI as a part of the ongoing active surveillance program for avian influenza viruses in Norway. Another two Eurasian wigeons shot in mid-November were found positive for influenza A virus of subtype H5N8, but sequencing of the cleavage site has not been successful.

Further, HPAI H5N8 was also detected in a Eurasian wigeon that was found dead in Randaberg municipality, Rogaland, in early December and a barnacle goose that was found dead in Evje and Hornnes municipality, Agder county, in mid-December (Fig. 1; Table 1).

## Discussion

The wetland areas in Jæren, in Rogaland county in south-western Norway (Fig. 1), are important habitats for migratory birds during the autumn, winter and spring roosts [8]. Jæren also has Norway’s highest density of poultry farms, which entails a risk for outbreaks of HPAI in laying hens and broilers [9]. It was thus not a surprise that the first detection of HPAI in Norway happened in Rogaland. However, the timing of HPAI virus introduction to Norway was more unforeseen, since the influx of potentially infected birds from central Europe during the spring migration was considered to pose the greatest risk to Norway [9].

The population of pink-footed geese that breed in the Arctic archipelago of Svalbard, migrate through Norway mainland in the autumn, from mid-September to late October [10]. The majority of the birds stage in Trøndelag county, where they are subject to regular hunting. After the staging period, they continue their migration southward, crossing inland to the Oslofjord and further to Denmark. Some birds, however, migrate along the western coast of Norway, and some stage in the wetlands of Jæren in Rogaland (Fig. 1). Most birds belonging to this population winter in the Netherlands and Belgium [10].

Observations from neck banded birds indicate regular short distance return migrations from other North Sea countries to Southern Norway [11]. In line with this, recent results from a goose fitted with a GPS-logger indicate that pink-footed geese may regularly move from Denmark to south-western Norway, especially in mild winters (Jesper Madsen, personal communication in Molværsmyr et al. [11]). Pink-footed geese breeding in Iceland or eastern Greenland winter in the United Kingdom [11]. However, a small number of pink-footed geese from these populations are occasionally found in Southern Norway [8, 12].

A seroprevalence of 47 % to avian influenza A virus was found in pink-footed geese sampled during the spring roost in Central Norway (Svalbard breeding population) between 2016 and 2018 [13]. Of the seropositive birds, 3 % (12/417) of the pink-footed geese had been exposed to the subtypes H5 and/or H7 [13]. Considering that HPAI viruses found in nature have almost always contained the H5 or H7 hemagglutinin, one may hypothesize that HPAI has circulated among pink-footed geese visiting Norway in previous years. The retrospective detection of four positive Eurasian wigeons, eight days *before* the first pink-footed goose, indicates that HPAI virus has been circulating in the Jæren area for some time, and also emphasizes the uncertainty about which species may have introduced the virus to Norway.

From the analyses of data deriving from counts of geese in Jæren, as well as neck band observations and ordinary recoveries of ringed birds [11], some potential introduction routes of the HPAI virus to Norway are discussed below.

Wild birds migrating from Russia to the south-west during autumn have been proposed as a potential pathway for the introduction of HPAI virus to Europe [14], but it is difficult to assess whether this may also apply to Norway because of the lack of data on bird migration from Russia to Norway.

Similarly, we cannot rule out that geese from Iceland brought the virus to Norway during autumn migration. However, HPAI virus has not been detected in Iceland and this route of introduction is considered less likely.

A northward return migration of geese from their wintering sites in areas extending from Denmark to Belgium, to sites in Southern Norway, has recently been documented [11]. Thus geese and other bird species may visit this region of Norway anytime during the winter season. Given that these birds have been in areas with outbreaks of avian flu during the autumn of 2020, such as the west coast of Denmark and the Netherlands [4], they may have introduced HPAI virus to Southern Norway.

The Barnacle goose, like the pink-footed goose, also migrates through Norway and breeds on Svalbard. It was the species with the highest number of reported cases of HPAI virus of subtype H5N8 in Europe during the autumn of 2020 [4]. Therefore, based on data by Molværsmyr et al. [11], we believe that HPAI virus of subtype H5N8 was most probably introduced by pink-footed geese and/or barnacle geese migrating to Norway from common night roosts along the west coast of Denmark, or from the Netherlands. However, more detailed studies of migratory routes of wild birds species, bird genetics [15] and molecular characterization of the H5N8 virus, are needed to answer this question. This is also illustrated by the four wigeons shot in mid-November, as it leaves an unanswered question of where and how these ducks were infected.

## Conclusions

HPAI virus has been detected for the first time in Norway, but the mode of introduction remains unclear. However, a northward migration of infected geese or gulls from Denmark or the Netherlands during the autumn of 2020, is currently our main hypothesis for the introduction of HPAI to Norway. More detailed studies of the migratory routes of wild birds species, bird genetics and molecular studies of H5N8 viruses, are needed to answer this question. The presence of HPAI in wild birds constitutes a new, and ongoing, threat to the Norwegian poultry industry, and compliance with the improved biosecurity measures on poultry farms should therefore be ensured. Finally, although HPAI of subtype H5N8 has been reported to have very low zoonotic potential, this is a reminder that HPAI with greater zoonotic potential in wild birds may pose a threat in the future. This underpins the importance of implementing a One Health strategy in handling avian influenza in Norway.

## Methods

The NFSA is responsible for implementing the active surveillance programme for avian influenza (AI) in wild birds in Norway. The programme started in 2005 and is based on screening of cloacal and oropharyngeal swabs from healthy birds shot during the hunting season [5]. Directly after sampling, swabs are placed in virus transport medium and mailed to the NVI in Oslo. Samples are either processed immediately or frozen at − 70 °C upon arrival. In addition, dead birds considered to be high risk species for HAPI (geese, ducks and gulls) are sampled in the field and submitted directly to NVI for PCR analysis [5].Virus detection is performed with real-time RT-PCR targeting the matrix gene of the Influenza A virus, followed by subtype-specific PCRs and sequencing of the cleavage site of the haemagglutinin molecule for determination of the pathogenicity, following the protocols from the Animal and Plant Health Agency (APHA) [16].

Preparation of DNA libraries were performed using the NexteraXT kit (Illumina, Cambridge, UK), indexed libraries were quantified using Quantifluor dsDNA System (Promega, Southampton, UK), pooled and sequenced on a NextSeq550 sequencing platform using a High Output Kit v2.5 with 2 × 150 base- paired end reads. These analyses were performed at APHA, Surrey, United Kingdom.

**Fig. 2:**
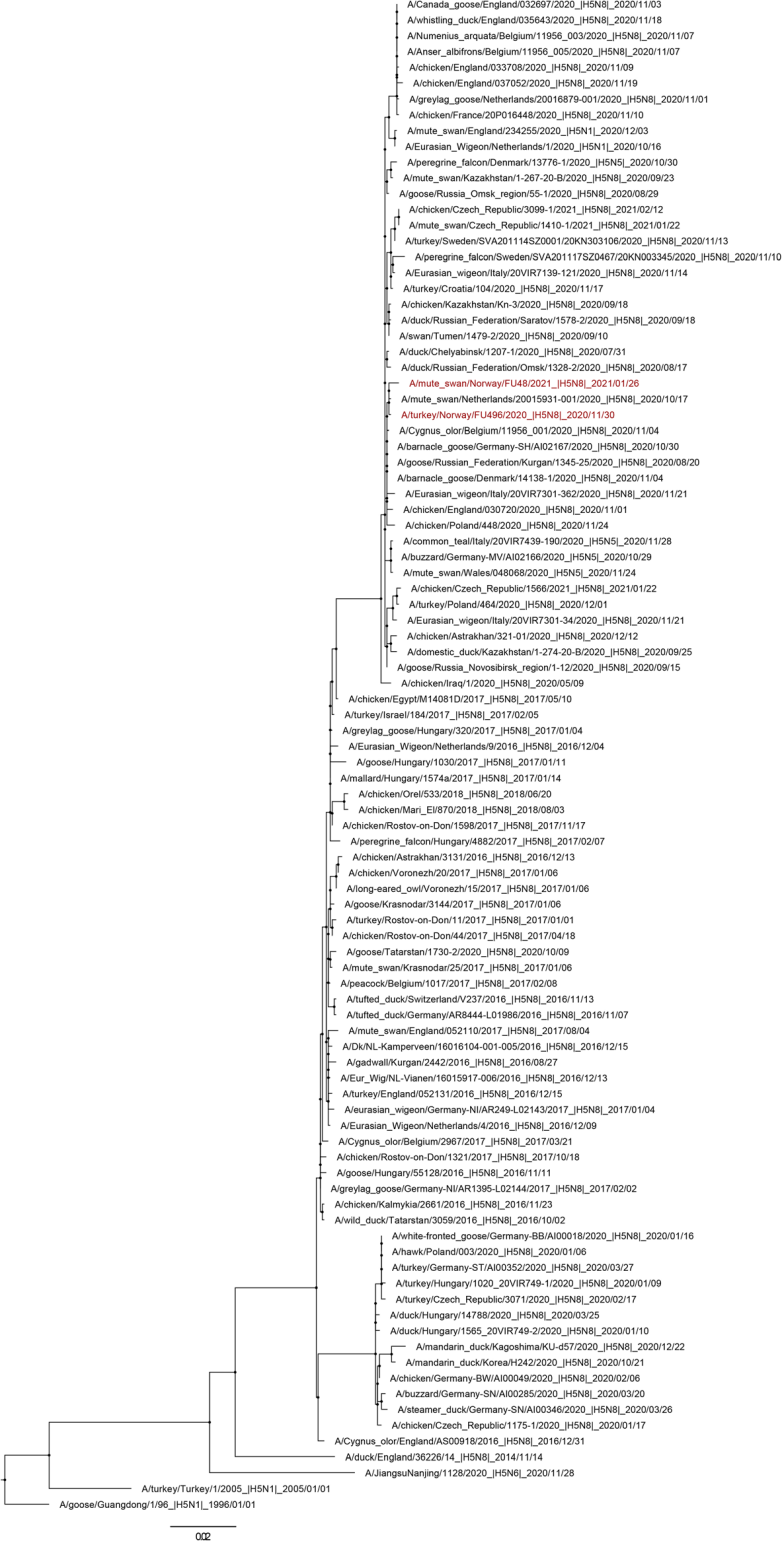
Maximum likelihood phylogenetic tree inferred from the HA gene segment of HPAI H5 viruses and rooted through the outgroup A/Goose/Guangdong/1/96. The HA gene sequences of HPAI H5N8 Norwegian isolates from 2020 to 2021 are coloured in red

Raw sequencing reads were assembled using a custom in-house pipeline, available on github (https://github.com/AMPByrne/WGS/blob/master/RefGuidedAlignment_Public.sh). Final consensus sequences were obtained by mapping to the genome of A/chicken/Iraq/1/2020 (EPI_ISL_623074). Closest genetic relatives on the GISAID EpiFlu database (https://platform.gisaid.org/ accessed on 25th February 2021) were identified using the BLAST function. The top 50 BLAST hits were downloaded and added to a manually curated alignment with related avian influenza virus HA sequences, the resultant H5Nx dataset was aligned using MAFFT v7.450 [17], inspected and trimmed to remove signal peptides and from the final stop codon with AliView [18]. We inferred a maximum likelihood phylogenetic tree for the HA segment using IQ-TREE v1.6.12 [19] and obtained branch supports with Shimodaira-Hasegawa (SH)-like approximate Likelihood Ratio Test (aLRT) with 1000 iterations. The tree was visualised and annotated using FigTree v1.4.3 (https://github.com/rambaut/figtree), rooted by A/Goose/Guangdong/1/96 and nodes were placed in ascending order (Fig. 2). Whole genome sequences were deposited on the GISAID database under accession numbers: EPI_ISL_1295638 and EPI_ISL_1295639.

## Data Availability

The datasets generated, used and analysed during the current study are available from the corresponding author on reasonable request.
